# Domain-Specificity of Creativity: A Study on the Relationship Between Visual Creativity and Visual Mental Imagery

**DOI:** 10.3389/fpsyg.2015.01870

**Published:** 2015-12-01

**Authors:** Massimiliano Palmiero, Raffaella Nori, Vincenzo Aloisi, Martina Ferrara, Laura Piccardi

**Affiliations:** ^1^Department of Life, Health and Environmental Sciences, University of L’Aquila, L’Aquila, Italy; ^2^Department of Psychology, University of Bologna, Bologna, Italy; ^3^Neuropsychology Unit, IRCCS Fondazione Santa Lucia, Rome, Italy

**Keywords:** creative cognition approach, imagery, transformation imagery, cognitive style, visualization strategy

## Abstract

Creativity refers to the capability to catch original and valuable ideas and solutions. It involves different processes. In this study the extent to which visual creativity is related to cognitive processes underlying visual mental imagery was investigated. Fifty college students (25 women) carried out: the Creative Synthesis Task, which measures the ability to produce creative objects belonging to a given category (originality, synthesis and transformation scores of pre-inventive forms, and originality and practicality scores of inventions were computed); an adaptation of Clark’s Drawing Ability Test, which measures the ability to produce actual creative artworks (graphic ability, esthetic, and creativity scores of drawings were assessed) and three mental imagery tasks that investigate the three main cognitive processes involved in visual mental imagery: generation, inspection and transformation. Vividness of imagery and verbalizer–visualizer cognitive style were also measured using questionnaires. Correlation analysis revealed that all measures of the creativity tasks positively correlated with the image transformation imagery ability; practicality of inventions negatively correlated with vividness of imagery; originality of inventions positively correlated with the visualization cognitive style. However, regression analysis confirmed the predictive role of the transformation imagery ability only for the originality score of inventions and for the graphic ability and esthetic scores of artistic drawings; on the other hand, the visualization cognitive style predicted the originality of inventions, whereas the vividness of imagery predicted practicality of inventions. These results are consistent with the notion that visual creativity is domain- and task-specific.

## Introduction

Creativity is a mysterious aspect of human thinking. The general characteristics shared by the creative products are hard to recognize. There is wide agreement on the notion that creativity involves the ability to produce a work that is both original and appropriate (Sternberg and Lubart, 1996; Mumford, 2003), leading to new inventions and solutions in any area (Vanderbos, 2006). Therefore, creativity plays a crucial role on human thought, being involved in different activities, such as problem-solving (Basadur et al., 2000), scientific progress (Klahr and Simon, 1999), verbal thought (Fink and Neubauer, 2006), visual art (van Leeuwen et al., 1999), dance (Fink et al., 2009), music (Olivetti Belardinelli, 2002), and so forth. This multi-componential aspect of creativity opens to the issue of domain-specificity. According to Hong and Milgram (2010) creativity can be distinguishable in both domain-general and domain-specific. A though creativity has been long considered only domain-general, different studies showed that creativity is also domain-specific at both the behavioral (Plucker and Beghetto, 2004; Kaufman and Baer, 2005; Palmiero et al., 2010) and neuroanatomical levels (Boccia et al., 2015). In general, following the psychometric approach of individual differences, various creativity-relevant skills, such as the tolerance for ambiguity (Amabile, 1996), and divergent thinking (Silvia, 2008), which involves the assessment of the creative potential rather than of the creative outcomes (Runco and Acar, 2012), play a key role on creativity across many different domains. On the contrary, following problem-solving theories creativity appears to be more domain-specific (Silvia et al., 2009). Interestingly, when the focus is on the creative product creativity is mostly domain-specific (Baer, 1993). Thus, besides the domain specificity, creativity production also depends on the features of the domain and approach used. For example, Palmiero et al. (2010) showed that visual creativity is more domain- and task-specific than verbal creativity.

In the present paper, the extent to which visual creativity is related to visual mental imagery was explored considering the product-oriented approach. Firstly, the creative cognition approach (Finke and Slayton, 1988; Finke, 1990, 1996; Finke et al., 1992) was used. It focuses on the mental operations supporting visual creativity rather than on individual differences (Abraham and Windmann, 2007). According to Finke, generative processes (e.g., association, mental synthesis) are used in the construction of pre-inventive forms, and exploratory processes (e.g., conceptual interpretation, functional inference) are used to examine and interpret the pre-inventive forms. Although this approach encompasses a strong visual imagery component, only a few studies revealed that specific dimensions of the creative objects production are related to specific visual imagery operations (e.g., Palmiero et al., 2011; Morrison and Wallace, 2001; Verstijnen et al., 1998), whereas other studies failed to find such a relationship (Anderson and Helstrup, 1993; Palmiero et al., 2010).

Secondly, the visual creative behavior approach was used. The actual creative behavior involving performance in visual arts and drawing is assumed to be related to vividness of imagery, which refers to the pictorial dimension of imagery, given that previous studies revealed that vividness of imagery (Pérez-Fabello and Campos, 2007) and the ability to generate mental images to identify letters with parts omitted (Zemore, 1995) are enhanced in the presence of artistic training. In this direction, Morrison and Wallace (2001) also found a positive relationship between vividness and creative behavior in art in psychology students with artistic background as assessed by the visual art sub-score of the Creative Behavior Inventory (Hocevar, 1979). In contrast, the extent to which the actual visual creative behavior is related to performances on tests that measure the ability to mentally manipulate spatial images in two- or three-dimensions is unclear, since different studies failed to find such a relationship (for a review, see Palmiero and Srinivasan, 2015). This is consistent with the idea that people with formal artistic training or with involvement in past visual art rely on object imagery, preferring to construct high-resolution images of objects of scenes, rather than represent spatial relations among visual elements (Blazhenkova and Kozhevnikov, 2009).

The relationship between visual creativity products and visual mental imagery was investigated within Kosslyn’s (1980) theoretical framework, which sustains that three different cognitive processes underlie visual imagery: generation of mental images of visual stimuli previously learned; inspection of visual details within a mental image, and transformation of abstract representations before matching them with visual stimuli. To this end, the Creative Synthesis Task (Finke, 1990), aimed at constructing creative objects, and Clark’s Drawing Ability Test (Clark, 1989), aimed at making artistic drawings, were used. In addition, besides the tasks of generation, inspection and transformation of images, vividness of imagery was also measured to investigate the ability to represent pictorially visual images, and the verbalizer–visualizer cognitive (VVQ) style to investigate a personal general attitude toward visual mental imagery. Given that the relationship between visual creativity and visual imagery has never been studied by such a combination of approaches, the present study is unique in this respect.

## Materials and Methods

### Participants

The research involved 50 College students from the “Department of Life, Health and Environmental Sciences,” University of L’Aquila, Italy: 25 women (mean age = 20.64 ± 1.32—age range = 19–24) and 25 men (mean age = 23.4 ± 4.20—age range = 19–31). All participants were healthy and without neurological and/or psychiatric disorders; no problem with alcohol or drug addiction was reported. None of the participants had a background in art or creative activities in general. In order to exclude those with significant visuo-spatial working memory problems, the Corsi Block-tapping Test (Corsi, 1972; for the test procedure, see Piccardi et al., 2013) was administered both forward and backward. None of the participants was found to be impaired in visuo-spatial working memory (Corsi forward: men mean = 6.12; SD = 1.20; cut-off < 4.68; women mean = 6.29, SD = 1.28; cut-off < 4.07 in Piccardi et al., 2013; Corsi backward: men mean = 5.84; SD = 1.11; cut-off < 3.44; women mean = 5.76, SD = 0.91; cut-off < 3.44 in Monaco et al., 2013). All participants had normal or corrected to normal (soft contact lenses or glasses) vision. Everyone signed the written informed consent after the procedures had been fully explained to them. The study was designed in accordance with the ethical principles of human experimentation stated in the Declaration of Helsinki and was approved by the Institutional Review Board of the Department of Life, Health and Environmental Science, University of L’Aquila.

### Materials and Procedure

Participants took part in the study individually. The experiment lasted approximately 2 h. The following tasks and questionnaires were administered in random order.

#### Creative Tasks:

***The creative synthesis task***

**FIGURE 1:**
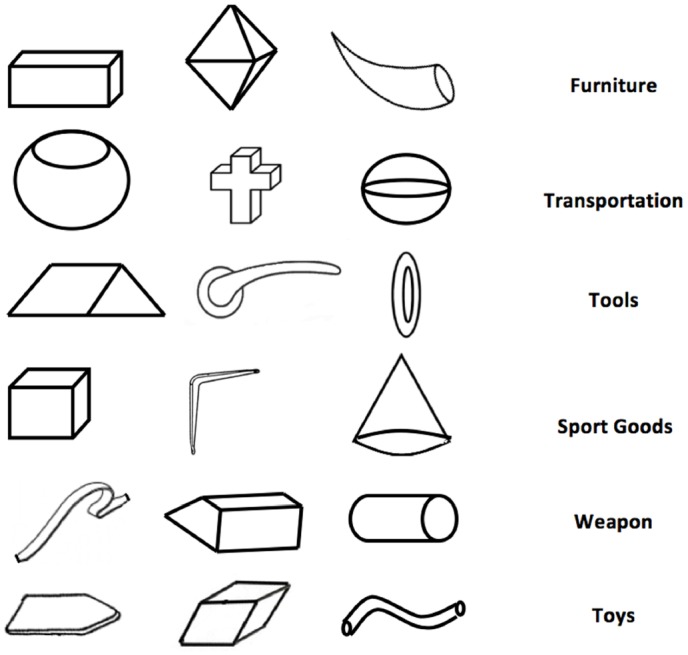
**Triads of Elements for the Figural Combination Task: (1) rectangular block, dipyramid, horn (furniture); (2) pot, cross, sphere (transportation); (3) rhombus, handle, ring (tools); (4) cube, bracket, cone (sport goods); (5) strip, trapezoid, cylinder (weapons); (6) board, rhomboid, tube (toys)**.

The Creative Synthesis Task (Finke, 1990) aimed to create objects belonging to specific categories, starting from visual components. Six triads of components and six categories were used (see Figure 1). The same combinations of stimuli and categories were presented across participants to increase the inter-rater reliability by reducing random error variation (Abraham and Windmann, 2007).

Participants were first introduced to the task with a practical example. Following Finke (1990), for each triad participants were given 2 min to mentally combine the components into a pre-inventive form (potential useful object). Components could be changed in position, rotation, and size, but not in their general structure. The instructions encouraged participants to assemble the visual components at their best and to sketch the pre-inventive form as they generated it. For each triad, names of stimuli were written in the upper part of a sheet of paper, and stimuli were drawn below. Participants were given 15 s to memorize the stimuli, and were then allowed to think of their pre-inventive form and subsequently sketch it on the sheet of paper. After creating the six pre-inventive forms, participants were presented with a category name for each of them and instructed to think of their objects as an invention within the category. Participants were given 1 min to describe the functioning of the invention and to write its name.

***Clark’s drawing ability test***

Clark’s Drawing Ability Test (Clark, 1989; Clark and Zimmerman, 2004) aimed to create in the visual arts. Participants were instructed to draw only two pictures out of four of the original version of this task: a front view of a house; a fantasy drawing from imagination. Participants were given 10 min per drawing. Colors were available. The instructions encouraged participants to be as creative as possible while making their artistic drawings.

#### Visual Imagery Testing

***Vividness and cognitive style tasks***

*The vividness of visual imagery questionnaire.* The Vividness of Visual Imagery Questionnaire (VVIQ; Marks, 1973) aimed to measure the vividness of visual imagery. Participants were instructed to rate 16 visual mental images cued by verbal descriptions along a 5 point-scale ranging from 1 (no image at all) to 5 (image clear and vivid as reality). The maximum score was 80.

*The verbalizer–visualizer questionnaire.* The Verbalizer–Visualizer Questionnaire (VVQ; Richardson, 1977) aimed to measure individual differences in the dimension of visualizer/verbalizer cognitive style, where visualizers were supposed to have high-imagery ability and verbalizers were supposed to have low imagery ability. Participants were instructed to choose a true/false response to 15 questions, such as “I like to learn new words” or “My dreams are extremely vivid.” The maximum score was 15.

**FIGURE 2:**
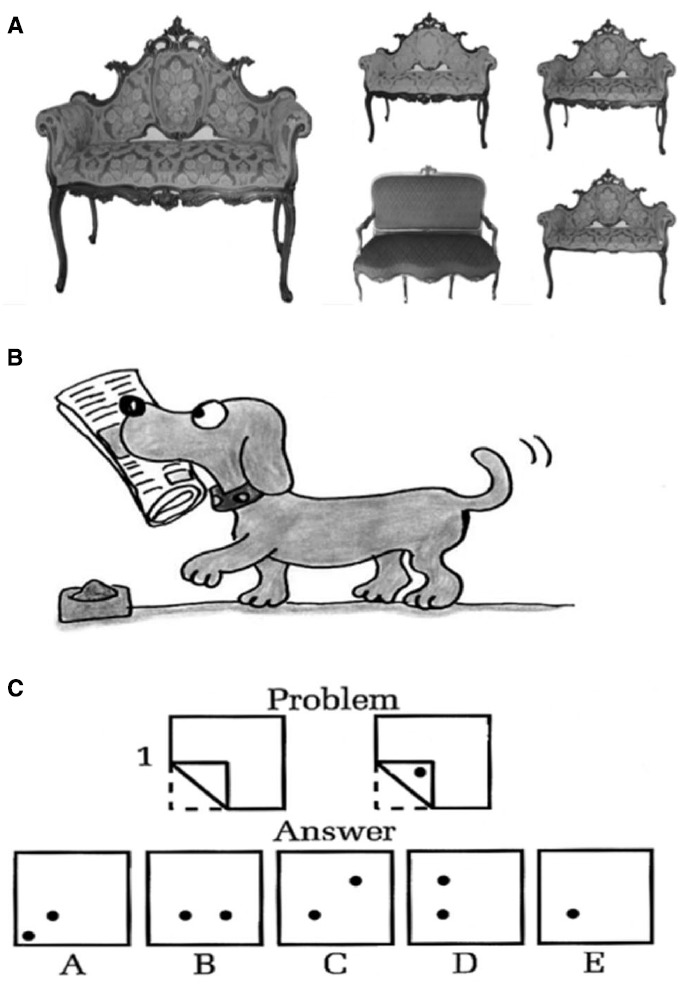
**(A)** Generation Task: Example of item of the Object task: target (on the left), response option (on the right). **(B)** Inspection Task: Example of item of the Object Inspection Task. **(C)** Transformation Task: Example of item of the Paper Folding Task. The figures “Problem” represent a square piece of paper being folded, and the last of these figures has one or two small circles drawn on it to show where the paper has been punched. Each hole is punched through all the thicknesses of paper at that point. The five figures “Answer” show where the holes will be when the paper is completely unfolded.

The tasks proceeded according to the three components of Kosslyn’s Model (see Figure 2).

#### Generation Task

***The object task***

The Object task (Palermo et al., 2010) is a generation task including 20 items, each of which consisted of two stimuli. In each item, the first stimulus was a photo of an object (e.g., a boot, a spoon, etc.). The participants were asked to observe the photo target for 10 s. They then had to mentally generate the image of the previously seen photo with their eyes closed. When the participant was ready, the second stimulus was shown. In the second stimulus four pictures of objects were presented: the target and three distractors (see Figure 2A). The distractors included a mirror image of the target and two objects similar to the target, but with different basic visual characteristics (i.e., color) or with modification (or cancelation) of specific elements (such as the high heel of a boot or the decorative elements in the handle of a spoon). The score was either 1 (correct) or 0 (incorrect); the highest score was 20.

#### Inspection Task

***The object inspection task***

The Object Inspection Task (Nori et al., 2012) is an inspection task in which participants observed a picture for 20 s. The picture was then removed and the participants were asked to close their eyes, to imagine the picture previously seen and to answer questions about it (i.e., Was the dog’s tail pointing up or down? see Figure 2B). The score was either 1 (correct) or 0 (incorrect), the highest score was 20.

#### Transformation Task

***The paper folding test***

The Paper Folding Test (PFT; Ekstrom et al., 1976) is normally used to measure spatial visualization ability, reflecting the ability to perceive, encode and mentally manipulate spatial forms (Lohman, 1988). It involves a strong imagery component, given that the performance on this task was found to be related to the ability to rotate and integrate imaged forms (Poltrock and Agnoli, 1986). Participants were presented with figures of papers being folded and holes being punched in the folded papers. They were instructed to imagine what the pattern of holes would look like if the paper were unfolded (see Figure 2C). Participants were given 6 min to complete the test, consisting of twenty items in total. The maximum score was 20.

### Data Scoring

Before carrying out statistical analyses, data were scored according to different criteria. Visual creativity tasks were evaluated using Amabile’s (1983) Consensual Assessment Technique, encompassing the idea that creativity products can be measured as the combined judgment of different people. Therefore, two independent and anonymous judges (as in other studies, e.g., Kavakli and Gero, 2001; Pearson et al., 2001; Abraham et al., 2006; Abraham and Windmann, 2008; Roskos-Ewoldsen et al., 2008; Palmiero, 2015), one female (23 years old) and one male (23 years old), were instructed with practical examples to evaluate both the pre-inventive forms and inventions created by participants by means of the Creative Synthesis Task (pre-inventive forms were rated independently of inventions) as well as the front view of a house and the fantasy drawing of Clark’s Drawing Ability Test. Unrecognizable drawings were excluded from the analysis. For each criteria described below the average of the ratings given by the judges was taken as the final score.

The pre-inventive forms of the Creative Synthesis Task were evaluated in terms of: “originality,” defined as a form being new and not derived from something else, from 1 (very poor originality) to 5 (very high originality); “synthesis,” defined as the extent to which components were well assembled together, from 1 (very poor synthesis) to 5 (very high synthesis); “transformation,” defined as the extent to which components were changed in position, orientation and size when assembled into the final form, from 1 (very poorly transformed) to 5 (very highly transformed). The inter-rater correlations (intra-class correlation coefficient—absolute agreement) were significant for both “originality” (α = 0.488, *p* < 0.05), “synthesis” (α = 0.546, *p* < 0.005), and “transformation” (α = 0.501, *p* < 0.01).

Inventions were evaluated in terms of: “originality,” defined as an invention being new and not derived from something else, from 1 (very poor originality) to 5 (very high originality); “practicality,” defined as an invention involving an actual use in a specific context, rather than a hypothetical use, from 1 (very poor practicality) to 5 (very high practicality). The inter-rater correlations (intra-class correlation coefficient—absolute agreement) were significant for both “originality” (α = 0.583, *p* < 0.001) and “practicality” (α = 0.481, *p* < 0.05).

The creative drawings were evaluated in terms of: “graphic ability,” defined as the extent to which drawings were performed accurately in terms of pictorial aspects, such as colors, shades, details provided, as well as in terms of spatial aspects, such as spatial relations among elements; “esthetic,” defined as the extent to which drawings involved beauty, giving pleasure and satisfaction viewing them; “creativity,” defined as drawings involving different new ideas, perspective, colourfulness. The inter-rater correlations (intra-class correlation coefficient—absolute agreement) were significant for “graphic ability” (α = 0.896, *p* < 0.001), “esthetic” (α = 0.815, *p* < 0.001), “creativity” (α = 0.746, *p* < 0.001).

Regarding the Visual Imagery Tasks, the Building task, the Object Inspection Task and the PFT were scored by summing the number of correct responses (max 20 for each task), in order to obtain one independent variable for each cognitive process involved in visual mental imagery: generation, inspection and transformation, respectively.

The vividness score was computed by summing the scores of each item; the VVQ was scored according to Richardson’ norms. The score was treated as a continuous independent variable, assuming that the higher the VVQ score was, the higher the degree of imagery ability.

## Results

In order to explore the relationship between visual creativity and visual mental imagery abilities Pearson correlations between different variables were performed (see Table 1). On the one hand, variables related to visual creativity were: “originality,” “synthesis,” “transformation” of pre-inventive forms; “originality” and “practicality” of inventions; “graphic ability,” “esthetic,” and “creativity” of Clark’s Drawing Ability Test. On the other hand, variables related to visual mental imagery were: accuracy of the generation, inspection and transformation processes of visual imagery, the vividness score of visual imagery (VVIQ), the degree of style VVQ.

**TABLE 1:** **Correlation matrix**.

	**VVIQ**	**VVQ**	**Gen-ACC**	**Isp-ACC**	**Transf-ACC**
Ori-Pre	–0,03	0,13	–0,05	0,14	0,37
Syn-Pre	–0,02	0,06	–0,05	0,04	0,38
Transf-Pre	–0,21	0,24	–0,04	0,08	0,32
Ori-Post	–0,06	0,32	0,02	0,07	0,41
Pract-Post	–0,33	0,24	0,22	0,09	0,30
GA-CDAT	0,02	0,26	0,05	0,09	0,48
Aesth-CDAT	–0,03	0,22	0,03	0,06	0,47
Creat-CDAT	0,09	0,17	–0,05	0,05	0,33

Correlations in Bold are significant. VVIQ, Vividness of Visual Imagery Questionnaire; VVQ, Verbalizer–Visualizer Questionnaire; Gen-ACC, Accuracy of Generation process; Insp-ACC, Accuracy of Inspection process; Transf-ACC, Accuracy of Transformation process; Ori-Pre, Originality of pre-inventive forms; Syn-Pre, Synthesis of pre-inventive forms; Transf-Pre, Transformation of pre-inventive forms; Ori-Inv, Originality of inventions; Pract-Inv, Practicality of inventions; GA-CDAT, Graphic Ability of the Clark’s Drawing Ability Task; Aesth-CDAT, esthetic of the Clark’s Drawing Ability Task; Creat-CDAT, Creativity of the Clark’s Drawing Ability Task.

The VVIQ score was found to be negatively correlated with the “practicality” score of inventions (*r* = -0.33; *p* < 0.05), whereas the VVQ score was positively correlated with the “originality” score of inventions (*r* = 0.32; *p* < 0.05). The PFT score was found to be positively correlated with all measures of creativity tasks, as follows: “originality” (*r* = -0.37; *p* < 0.01), “synthesis” (*r* = -0.38; *p* < 0.01), and “transformation” (*r* = -0.32; *p* < 0.05) scores of pre-inventive forms; “originality” (*r* = -0.41; *p* < 0.01) and “practicality” (*r* = -0.30; *p* < 0.05) scores of inventions; “graphic ability” (*r* = -0.48; *p* < 0.001), “esthetic” (*r* = -0.47; *p* < 0.005), and “creativity” (*r* = -0.33; *p* < 0.05) scores of Clark’s Drawing Ability Test.

In addition, Hierarchical Regression analyses aimed at investigating the extent to which visual creativity scores can be predicted by the vividness of imagery, the VVQ style and mental imagery abilities were performed. A Hierarchical Multiple Regression analysis was carried out for each of the following dependent variables: “originality,” “synthesis,” and “transformation” scores of pre-inventive forms; “originality” and “practicality” scores of inventions; “graphic ability,” “esthetic,” and “creativity” scores of Clark’s Drawing Ability Test. For all analyses the predictor “gender” was first entered in order to check for any difference; then the VVIQ and VVQ scores were entered, followed by the accuracy of the generation, inspection and transformation processes. In total, three blocks of independent variables were used.

The analysis showed that the overall model of the “originality” [*F*_(6,43)_ = 1.423, *p* > 0.05], “synthesis” [*F*_(6,43)_ = 1.35, *p* > 0.05] and “transformation” scores [*F*_(6,43)_ = 1.841, *p* > 0.05] of pre-inventive forms were not significant.

Regarding the “originality” score of inventions, the analysis demonstrated that the first model was not significant [*F*_(1,48)_ = 1.341, *p* > 0.05]. After introducing the VVIQ and VVQ scores, the second model explained 17.7% of variance [*F*_(3,46)_ = 3.287, *p* < 0.05; R^2^ = 0.177; R^2^ Adjusted = 0.123], that is an additional 15% [R^2^ change = 0.149; *F*_(2,46)_ = 4.171; *p* < 0.05]. After introducing the accuracy score of the generation, inspection and transformation processes of visual mental imagery, the third model explained 31.5% of variance [*F*_(6,43)_ = 3.295 *p* < 0.01; R^2^ = 0.315; R^2^ Adjusted = 0.219], that is an additional 13.8%, [R^2^ change = 0.138; *F*_(3,43)_ = 2.897; *p* < 0.05]. In the model as a whole, the VVQ score (β = 0.420, *p* < 0.01, *t* = 2.919) and the PFT score (β = 0.325, *p* < 0.05, *t* = 2.408) were significant.

Regarding the “practicality” score of inventions, the analysis showed that the first model was not significant [*F*_(1,48)_ = 0.0, *p* > 0.05]. After introducing the VVIQ and VVQ scores, the second model explained 17.1% of variance [*F*_(3,46)_ = 3.157, *p* < 0.05; R^2^ = 0.238; R^2^ Adjusted = 0.170], that is an additional 17.1% [R^2^ change = 0.171; *F*_(2,46)_ = 4.736, *p* < 0.01]. After introducing the accuracy score of the generation, inspection and transformation processes of visual mental imagery, the third model explained 29.2% of variance [*F*_(6,43)_ = 2.962, *p* < 0.05; R^2^ = 0.292; R^2^ Adjusted = 0.194], that is an additional 12.1% [R^2^ change was not significant, *F*_(3,43)_ = 2.466, *p* > 0.05]. In the model as a whole, only the VVIQ score (β = –0.307, *p* < 0.05, *t* = –2.311) was significant.

Regarding the “graphic ability” score of Clark’s Drawing Ability Test, the first [*F*_(1,48)_ = 0.334, *p* > 0.05] and the second [*F*_(3,46)_ = 1.114, *p* > 0.05] model were not significant. After introducing the accuracy score of the generation, inspection and transformation processes of visual mental imagery, the third model explained 30.5% of variance [*F*_(6,43)_ = 3.146, *p* < 0.05; R^2^ = 0.305; R^2^ Adjusted = 0.208], that is an additional 23.7%, [R^2^ change = 0.237; *F*_(3,43)_ = 4.895, *p* < 0.05]. In the model as a whole, only the PFT score (β = 0.479, *p* < 0.001, *t* = 3.515) was significant.

Regarding the “esthetic” score of Clark’s Drawing Ability Test, the first [*F*_(1,48)_ = 0.975, *p* > 0.05] and the second [*F*_(3,46)_ = 0.950, *p* > 0.05] model were not significant. After introducing the accuracy score of the generation, inspection and transformation processes of visual mental imagery, the third model explained 28.9% of variance [*F*_(6,43)_ = 2.908, *p* < 0.05; R^2^ = 0.305; R^2^ Adjusted = 0.208], that is an additional 23%, [R^2^ change = 0.230; *F*_(3,43)_ = 4.641, *p* < 0.05]. In the model as a whole, only the PFT score (β = 0.494, *p* < 0.001, *t* = 3.587) was significant.

Regarding the “creativity” score of Clark’s Drawing Ability Test, the analysis showed that the overall model [*F*_(6,43)_ = 1.305, *p* > 0.05] was not significant.

## Discussion

This study was aimed at investigating the extent to which dimensions of visual creativity, measured in terms of creative objects production and artistic drawings making, and imagery components, such as vividness of imagery, the strategy to use preferentially images and cognitive processes involved in imagery (generation, inspection and transformation of images) are related. The correlation analysis revealed that all dimensions of pre-inventive forms, inventions and artistic drawings positively correlated with the transformation imagery ability measured by means of the PFT, meaning that the higher the ability to construct pre-inventive forms, original and practical inventions and to make creative artistic drawings, the higher the ability to mentally manipulate spatial forms. According to our results only the high capability to mentally transform an image predicts dimensions of visual creativity products. Among the three cognitive components of visual mental imagery, transformation is the only one that requires a high cognitive load also involving working memory. All participants enrolled in the study showed a working memory capability above the cut-off reported by the two most recent Italian validation studies (Piccardi et al., 2013; Monaco et al., 2013) of the Corsi Test, and we can therefore exclude that this result could be a consequence of other cognitive processes underlying visual mental imagery transformation.

Focusing on the Creative Synthesis Task, the construction of pre-inventive forms, based on mental transformations and syntheses of visual elements, as well as the interpretation of pre-inventive forms were supported by the spatial imagery ability. These results confirm and extend Roskos-Ewoldsen et al. (2008), who found relationships between the originality score of pre-inventive forms and inventions and the PFT score in a sample of 70 people composed of young and old people. In addition, they are also in line with Morrison and Wallace (2001), who found significant correlations between different measures of rated creativity and recognisability of objects and the Surface Development Test (Ekstrom et al., 1976), which measures the ability to mentally assemble three-dimensional shapes in order to match lettered edges with numbered edges on an assembled shape. In other words, the creative object production involves mental transformations that comply with the way spatial forms are assembled, and pre-inventive forms are also explored, probably using analogical reasoning to determine the creative value of objects.

The regression analysis partially confirmed these results, given that the transformation imagery ability only predicted the originality score of inventions. Interestingly, the originality score of inventions positively correlated with the Visualizer-Verbalizer score: the higher the ability to use images, the more original were the inventions in the Creative Synthesis Task. This result was also confirmed by the regression analysis. Therefore, both the spatial imagery ability and the strategy to use images to process information play a key role when interpreting pre-inventive meaningless forms. In other words, while searching for a creative object in the category, participants probably mentally visualized possible objects of the same shape as the pre-inventive form and used spatial imagery to compare shapes.

In addition, the practicality score of inventions was found to be negatively correlated with the vividness of imagery, meaning that the more participants imagine vividly the less practical were their inventions. These results were also confirmed by the regression analysis. This apparently contradicts Palmiero et al. (2011), who found a positive correlation between the vividness score and the practicality score of inventions. However, this could be explained by taking into account the differences in the procedure between the two studies. In fact, Palmiero et al. (2011) used a one-step procedure, priming participants with object category names while performing on the Creative Synthesis Task, whereas in the present study a two-step procedure was used, that is participants were firstly instructed to construct pre-inventive forms and then interpret them within a specific conceptual category. One explanation might be that, if the category is not primed in advance, the ability to imagine pictorially is not useful while thinking of the practical use of objects, it likely being more important to classify objects in specific categories (Laws, 2002), regardless of the practical value. On the contrary, knowing the category in advance, as occurred in Palmiero et al. (2011), probably leads participants with high vividness to assemble objects thinking of the practical value, with positive effects on the relationships between the vividness and practicality dimension of objects.

Moving to Clark’s Drawing Ability Test, the graphic ability, the esthetic and creativity scores of drawings correlated only with the transformation imagery ability. The regression analysis confirmed the predictive role of the PFT for the graphic ability and esthetic scores of the artistic drawing. No relationship was found between dimensions of the artistic drawing and processes supporting object imagery, such as vividness of imagery. Although these results partially confirm Morrison and Wallace (2001), who found a correlation between the visual art sub-score of the Creative Behavior Inventory (Hocevar, 1979) and performance on the Surface Development Task, they contradict previous studies showing that artists rely more on object imagery rather than on spatial imagery (e.g., Kozhevnikov et al., 2005; Blajenkova et al., 2006; Pérez-Fabello and Campos, 2007). Moreover, the present result also contradicts Kozhevnikov et al. (2013), who found that the spatial visualization ability measured by means of different tests, including the PFT, loaded on the factor of scientific creativity measures, whereas object imagery ability loaded on the factor of artistic creativity measures, such as Torrance’s (1972) picture completion task and the Creative Behavior Inventory subscale of art achievement. However, it should be noted that in the present study novices and not experts were used. Thus, besides the expertise issue, the contrasting results are also consistent with the notion that the relationships between artistic creativity and imagery are also sensitive to the tasks used.

In conclusion, this study underlines that the relationship between visual creativity and visual mental imagery is rather problematic and hard to predict. Visual creativity is definitively supported by specific visual imagery processes, and this would led one to suppose that it is enabled by abilities that fall in the visual domain of knowledge, but the extent to which visual imagery processes play a key role seems to be task- and expertise-dependent. Results might change depending on the tasks used both to measure visual creativity and visual imagery proficiencies, as well as the individual differences in visual creativity and visual imagery processing. In the present study visual creativity was assessed in light of two different product-oriented tasks, and scores obtained were related to specific visual imagery abilities scores. Of course, given the complexity of the visual domain, the methodology used does not encompass the variety of possibilities that the relationships between visual creativity and visual imagery can take on. In this direction, it would be interesting to better consider the spatial domain. According to Gardner (1983, 1993), creativity in a specific domain relies on domain-specific intelligences. Thus, spatial intelligence would offer a unique opportunity to understand domain-specific creativity, as well as general creativity, given that this intelligence is not limited to visual domains (Gardner, 1983). Future studies should explore these relationships using different methodologies, including also variables relying on the creative person and divergent thinking, that are more domain-general aspects of creativity (Silvia et al., 2009). This would also help to clarify the extent to which domain-general abilities (e.g., divergent thinking, spatial abilities) affect visual creativity. Yet, it should be noted that the results discussed above were partially confirmed by the regression analysis. This may be due to several reasons, for example the statistical power of the analyses given the relatively limited number of subjects. Finally, only two independent judges were used to evaluate the Creative Synthesis Task (pre-inventive forms and inventions) and drawings of Clark’s Drawing Ability Test. Therefore, although the correlations proved statistically significant, the magnitude of these effects was moderate, and caution should be taken before drawing any definitive conclusion.

### Conflict of Interest Statement

The authors declare that the research was conducted in the absence of any commercial or financial relationships that could be construed as a potential conflict of interest.
